# MicroRNAs as Biomarkers for the Diagnosis of Ankylosing Spondylitis: A Systematic Review and Meta-Analysis

**DOI:** 10.3389/fmed.2021.701789

**Published:** 2021-08-10

**Authors:** Jiangbi Li, Xiaoping Xie, Weibing Liu, Feng Gu, Ke Zhang, Zilong Su, Qiangqiang Wen, Zhenjiang Sui, Pengcheng Zhou, Tiecheng Yu

**Affiliations:** ^1^Department of Orthopedics, The First Hospital of Jilin University, Changchun, China; ^2^Department of Orthopedics, The First People's Hospital of Yunnan Province, Kunming, China

**Keywords:** ankylosing spondylitis, microRNA, biomarkers, systematic review, meta-analysis

## Abstract

**Background:** Abnormal expression levels of microRNAs (miRNAs) were observed in ankylosing spondylitis (AS) in recent articles, suggesting that miRNAs may be used as biomarkers for AS diagnoses. In this paper, we conducted a meta-analysis to identify the overall diagnostic accuracy of miRNA biomarkers in AS patients.

**Methods:** An extensive search was undertaken in PubMed, Embase, Cochrane databases, and Wan Fang database up to 30 December 2020 using the following key words: (“microRNAs” or “microRNA” or “miRNA” or “miR” or “RNA, Micro” or “Primary MicroRNA”) and (“Spondylitis Ankylosing” or “Spondyloarthritis Ankylopoietica” or “Ankylosing Spondylarthritis” or “Ankylosing Spondylarthritides” or “Spondylarthritides Ankylosing” or “Ankylosing Spondylitis”) and (“blood” or “serum” or “plasma”). Statistical evaluation of dysregulated miRNAs using the sensitivity, specificity, positive likelihood ratio (PLR), negative likelihood ratio (NLR), diagnostic odds ratio (DOR), and the area under the curve (AUC).

**Results:** Twenty-nine articles reporting on the miRNAs of AS were included. A total of 42 miRNAs were observed to be up-regulated and 45 miRNAs were down-regulated in the AS cases compared with the controls. Besides, 29 studies from nine articles were included in our meta-analysis. The pooled sensitivity, specificity, PLR, NLR, DOR, and AUC were 0. 76 (95% CI, 0.70–0.81), 0.80 (95% CI, 0.74–0.85), 3.75 (95% CI, 2.82–5.01), 0.30 (95% CI, 0.24–0.39), 12.32 (95% CI, 7.65–19.83), 0.85 (95% CI, 0.81–0.88), respectively, suggesting a good diagnostic accuracy of miRNAs for AS.

**Conclusions:** Circulating miRNAs are deregulated in AS patients. miRNAs may be used as a relatively non-invasive biomarkers for the detection of AS.

## Introduction

Ankylosing spondylitis (AS) is a common chronic rheumatic disease. It is usually characterized by inflammatory back pain, structural and functional disorders, and restricted motion of spine (1). The prevalence of AS is ~0.32% in North America, 0.24% in Europe, 0.17% in Asia, 0.10% in Latin America, and 0.07% in Africa (2). Until now, AS remains difficult to diagnose before sacroiliac joint damage is visible on X-ray. In addition, the pathogenesis of AS is not fully understood yet. Thus, the diagnosis and treatment of this disease still remain difficult. Although AS is genetically closely related to human leukocyte antigen (HLA)-B27, in people of European descent, about 85% of AS patients are HLA-B27 positive (3). However, HLA-B27 has low specificity for AS, and only 5~6% of people with HLA-B27 will suffer from AS disease (4). Besides, HLA-B27 only accounts for <30% of the overall genetic risks of the pathology of AS (5). As a result, there is an urgent need for early biomarkers for AS diagnosis. In addition, there have also numerous other associated genetic polymorphisms been identified, including HLA class I and II alleles (6), the human major histocompatibility complex class I chain-related gene A (7), non-major histocompatibility complex (MHC) regions (8) and microRNAs gene (9), and so on.

MicroRNA (miRNA) is a highly conserved noncoding RNA oligonucleotide during evolution, which can regulate the expression of target genes at the post-transcriptional level. It was validated that miRNAs can regulate cell metabolism, differentiation, proliferation, apoptosis, etc. (10). The expressions of miRNAs can be changed under physiological and pathological processes or treatment (11). Moreover, miRNAs can be detected in a variety of sources, including blood, tissue and body fluids. miRNAs have a high accuracy for the diagnosis of osteosarcoma (12), cancers of central nervous system (13), colorectal cancer (14), and glioma (15). Over the past years, the diagnosis accuracy of circulating miRNAs for early detection of AS have been reported in multiple articles. However, some results were inconsistent due to the difference in sample size, study design, and type of specimen. To date, there is no comprehensive meta-analysis on this topic. Therefore, we conducted such meta-analysis to identify the overall diagnostic accuracy of miRNA biomarkers in AS patients.

## Methods

### Literature Search Strategy

PubMed, Embase databases, Cochrane databases, and Wan Fang database for relevant articles published before 30 December 2020 were searched using the following key words: (“microRNAs” or “microRNA” or “miRNA” or “miR” or “RNA, Micro” or “Primary MicroRNA”) and (“Spondylitis Ankylosing” or “Spondyloarthritis Ankylopoietica” or “Ankylosing Spondylarthritis” or “Ankylosing Spondylarthritides” or “Spondylarthritides Ankylosing” or “Ankylosing Spondylitis”) and (“blood” or “serum” or “plasma”). Only English and Chinese articles were involved.

### Article Selection

All included articles had to meet the following criteria: (1) all the patients reported must be diagnosed as AS; (2) the articles are aimed at identifying the overall accuracy of miRNAs in AS patients; (3) miRNAs for the diagnosis of AS were compared with the diagnosis of AS according to the modified New York criteria or the AS classification criteria; (4) primary outcomes were the fold-change, *p*-value, and the sensitivity, specificity, PLR, NLR, DOR, and AUC of miRNAs for AS; (5) the articles are either a retrospective or prospective observational design (for prospective design, we used baseline data). The criteria for exclusion were as follows: (1) duplicated articles; (2) case reports, reviews, meta-analyses, editorials, and letters; (3) insufficient data; (4) papers conducted in animals or cell lines.

### Data Extraction, Meta-Analysis, and Quality Assessment

Data extraction was performed as follows: last name of the first author, publication year; ethnicity of patients; miRNA expression profiling; sample numbers; sample types; direction of expression difference (up or down regulated); sensitivity and specificity data, fold-change from miRNA expression profile. Studies identified the sensitivity and specificity of individual miRNA about the diagnosis of AS were included in the meta-analysis, and the qualities of selected articles were further assessed using the modified version of the QUADAS-2 tool (16). Moreover, the expression analysis of a specific miRNA in articles is considered to be an independent study.

### Statistical Analysis

All statistical analyses were performed using Review Manager 5.3 and Stata 12.0. *P* < 0.05 and fold change ≥ 2 or ≤ 0.5 indicated statistical significance. Studies identified the sensitivity and specificity of individual miRNA about the diagnosis of AS were included in the meta-analysis. Several common evaluation indicators, including sensitivity, specificity, PLR, NLR, DOR, and AUC value, were used to evaluate the diagnostic accuracy of miRNAs. The between-study heterogeneity was evaluated with the *I*^2^ and χ^2^ statistics, *I*^2^ > 50% indicated significant heterogeneity; hence, a random-effect model was subsequently used to pool these results. Subgroup analysis and meta-regression analysis were used to identify sources of heterogeneity. Finally, Deeks' funnel plot analysis was performed to explore the potential publication bias.

## Results

### Included Articles

**Figure 1:**
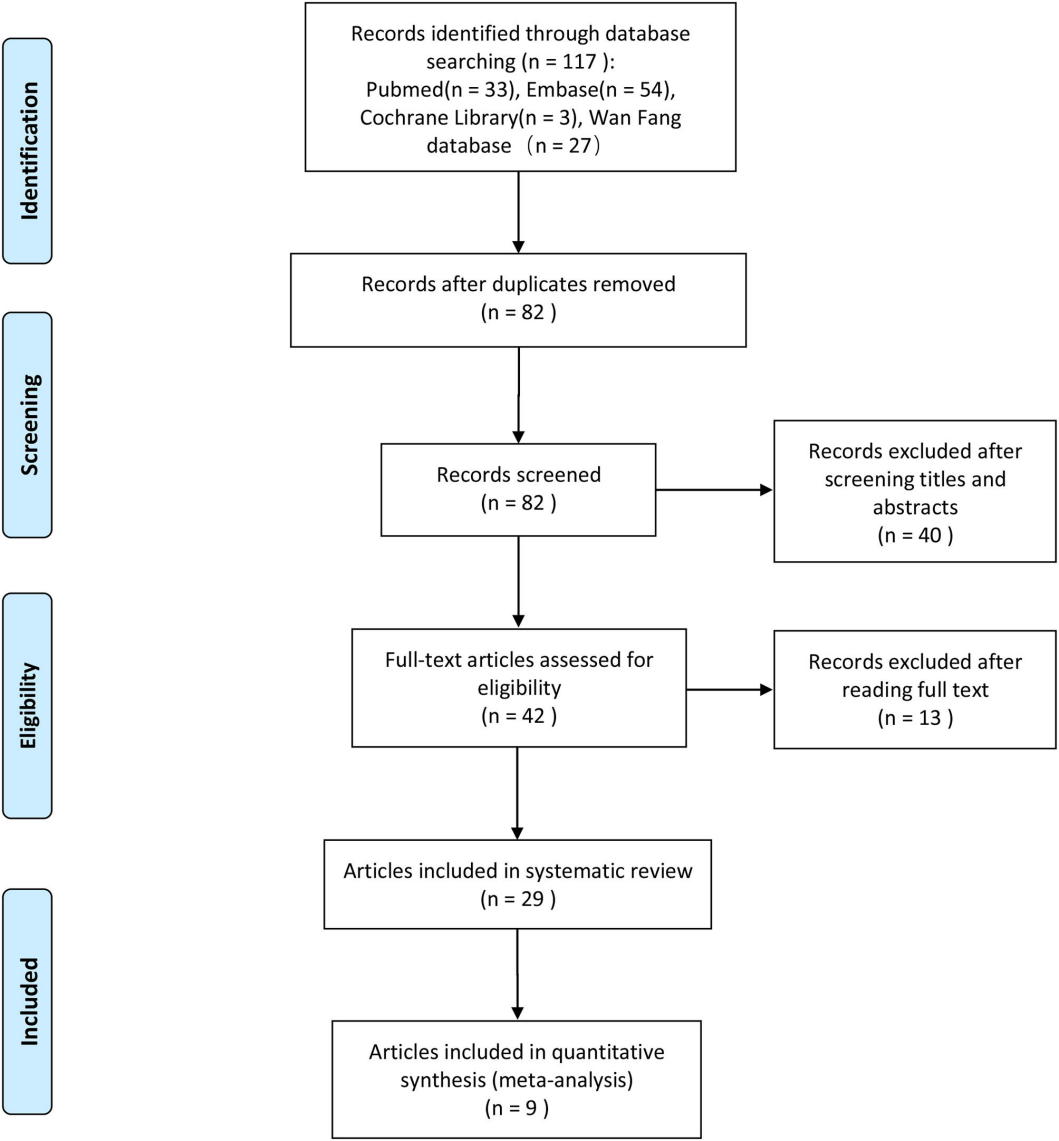
Flow diagram of article selection.

We initially identified 117 potentially eligible articles after the literature search process, and after removing 35 duplicated articles, 82 articles remained. In which, we excluded 40 articles after reading the titles and abstracts. And after full-text assessment, we eventually identified 29 articles that could be used for the systematic review of the miRNA expression profile. The full text of the 29 articles was assessed again in details, and nine of them (17–25) were included in our meta-analysis. A flowchart of the selection process of the articles is presented in Figure 1.

### Systematic Review of miRNAs Expression Profiles

A summary of 29 articles reporting on the miRNA expression profiles is shown in Table 1. A total of 87 miRNAs were differentially expressed between AS patients and healthy controls in these screened articles, and of which, 42 miRNAs were found to be up-regulated and 45 miRNAs were down-regulated in the AS cases compared with the controls. Twelve up-regulated miRNAs (miR-29, miR-146a, miR-126-3p, miR-155, miR-21, miR-214, miR-16, miR-181a, miR-223, miR-146a-5p, miR-151a-3p, and let 7i) and two down-regulated miRNAs (miR-17, miR-495) were identified by more than one studies, miR-29 was the most frequently identified dysregulated miRNA. Seven miRNAs (miR-29, miR-214, miR-16, miR-146a-5p, miR-151a-3p, miR-146a, and miR-223) were found to be either up-regulated or down-regulated in the AS cases as compared with the controls.

**Table 1:** Characteristics of studies included in systematic review.

**Study**	**Year**	**Country**	**Patients/controls**	**miRNAs profiled**		**Fold-change**	***P-*values**	**Specimen**
				**miRNA**	**Direction**			
Jin et al. (26)	2016	South Korea	3/3	miR-424-5p	**↑**	2.158072	≤ 0.05	PBMCs
				miR-136	**↓**	0.099969	≤ 0.05	
				miR-376a	**↓**	0.184359	≤ 0.05	
				miR-377	**↓**	0.21975	≤ 0.05	
				miR-29c*	**↓**	0.219023	≤ 0.05	
				miR-376c	**↓**	0.250179	≤ 0.05	
Turkyilmaz et al. (27)	2020	Turkey	50/50	miR-143	**↑**	1.4977	0.025721	PBMCs
				miR-142-5P	**↑**	3.3425	0.000003	
Wang et al. (28)	2017	China	40/40	miR-31	**↑**	–	0.001	PBMCs
Wei et al. (29)	2017	China	45/30	miR-146a	**↑**	–	≤ 0.01	PBMCs
Huang et al. (30)	2014	China	30/30	miR-29a	**↑**	–	≤ 0.001	PBMCs
Xia et al. (31)	2015	China	23/23	miR-124	**↑**	–	0.0017	Whole blood
Jiang et al. (32)	2015	China	20/20	miR-130a	**↓**	–	≤ 0.01	PBMCs
Qian et al. (33)	2016	China	10/10	miR-146a	**↑**	1.7	0.0016	Blood (serum)
				miR-155	**↑**	3.2	0.0058	
Huang et al. (1)	2014	China	122/122	miR-21	**↑**	–	0.012	Whole blood
Zhang et al. (34)	2018	China	50/25	miR-16a	**↑**	–	<0.05	Whole blood
Ciechomska et al. (35)	2018	Poland	13/15	miR-5196	**↑**	–	<0.001	Blood (serum)
Liu et al. (17)	2020	China	32/24	miR-214	**↑**	–	<0.001	Blood (serum)
Ni and Leng (18)	2020	China	150/150	miR-495	**↓**	–	<0.01	PBMCs
				miR-495	**↓**	–	<0.01	Whole blood
				miR-495	**↓**	–	<0.01	Blood (serum)
Reyes-Loyola et al. (36)	2019	Mexico	15/13	let-7i	**↑**	–	0.033	Blood (plasma)
Perez-Sanchez et al. (19)	2018	Spain	53/57	miR-320e	**↑**	3.0414	<0.05	Blood (plasma)
				miR-22-3p	**↑**	2.1703	<0.001	
				miR-146a-5p	**↑**	2.7578	0.001	
				miR-125a-5p	**↑**	2.665	<0.001	
				miR-151a-3p	**↑**	2.279	<0.001	
				miR-16-5p	**↓**	−2.262	–	
				miR-144-3p	**↓**	−3.628	–	
				miR-451a	**↓**	−5.151	0.043	
				miR-150-5p	**↓**	−3.461	0.045	
Lai et al. (37)	2013	China	22/18	let-7i	**↑**	–	0.004	Blood (T cells)
				miR-16	**↑**	–	0.02	
				miR-221	**↑**	–	0.032	
Lv et al. (38)	2015	China	40/50	miR-29a	**↓**	1/16.22	0.002	PBMCs
				miR-126-3p	**↓**	1/3.76	0.046	
Wang et al. (39)	2017	China	41/36	miR-199a-5p	**↓**	–	<0.05	Blood (T cells)
Kook et al. (40)	2018	Korea	65/39	miR-214	**↓**	–	<0.001	Blood (serum)
Magrey et al. (41)	2016	USA	15/5	miR-34a	**↑**	2.74	<0.05	Blood (plasma)
				miR-32	**↑**	2.26	<0.05	
				miR-16	**↓**	−3.13	<0.05	
				miR-10b	**↓**	−2.05	<0.05	
				miR-150	**↓**	−2.55	<0.05	
				miR-30a	**↓**	−2.26	<0.05	
				miR-154	**↓**	−2.20	<0.05	
Prajzlerova et al. (42)	2017	Switzerland	24/29	miR-374a-5p	**↓**		<0.001	Blood (plasma)
				miR-409-3p	**↓**	–	<0.001	
				miR-625-3p	**↓**	–	<0.001	
				miR-222-3p	**↓**	–	<0.001	
				miR-27a-3p	**↓**	–	<0.001	
				miR-29a-3p	**↓**	–	<0.001	
				miR-99b-5p	**↓**	–	<0.001	
				miR-146a-5p	**↓**	–	<0.01	
				miR-146b-5p	**↓**	–	<0.01	
				miR-221-3p	**↓**	–	<0.01	
				miR-223-3p	**↓**	–	<0.01	
				miR-106a-5p	**↓**	–	<0.01	
				miR-19a-3p	**↓**	–	<0.01	
				miR-24-3p	**↓**	–	<0.01	
				miR-151a-3p	**↓**	–	<0.05	
				miR-133a-3p	**↓**	–	<0.05	
				miR-140-3p	**↓**	–	<0.05	
				miR-145-5p	**↓**	–	<0.05	
Li et al. (20)	2019	China	43/39	miR-17-5p	**↑**	4.56	<0.01	PBMCs
				miR-27a	**↑**	2.81	<0.01	
				miR-29a	**↑**	7.15	<0.01	
				miR-126-3p	**↑**	4.09	<0.01	
Guo et al. (21)	2018	China	218/113	miR-132	**↑**	1.4977	<0.05	PBMCs
Jiang et al. (22)	2018	China	60/60	miR-17	**↓**	–	<0.01	PBMCs
				miR-17	**↓**	–	<0.01	Blood (plasma)
Jiang et al. (23)	2018	China	60/30	miR-146a	**↑**	–	<0.05	Blood (plasma)
				miR-155	**↑**	–	<0.05	
				miR-181a	**↑**	–	<0.05	
				miR-223	**↑**	–	<0.05	
				miR-146a	**↑**	–	<0.05	PBMCs
				miR-155	**↑**	–	<0.05	
				miR-181a	**↑**	–	<0.05	
				miR-223	**↑**	–	<0.05	
Huang et al. (43)	2019	China	20/20	miR-29	**↑**	–	0.00003857	PBMCs
Danaii et al. (44)	2017	China	35/40	miR-146a	**↓**	–	<0.0001	PBMCs
				miR-223	**↓**	–	=0.0025	
				miR-21	**↑**	–	=0.0018	
Liu (24)	2015	China	61/31	miR-181	**↓**	–	<0.05	PBMCs
				miR-495	**↓**	–	<0.05	
Fotoh et al. (25)	2020	Egypt	55/55	miR-125a	**↑**	–	<0.001	Blood (plasma)
				miR-451a	**↓**	–	<0.001	

*↑, up regulated; ↓, down regulated; –, not reported; PBMCs, peripheral blood mononuclear cells*.

### Article Characteristics in the Meta-Analysis and Quality Assessment

**Figure 2:**
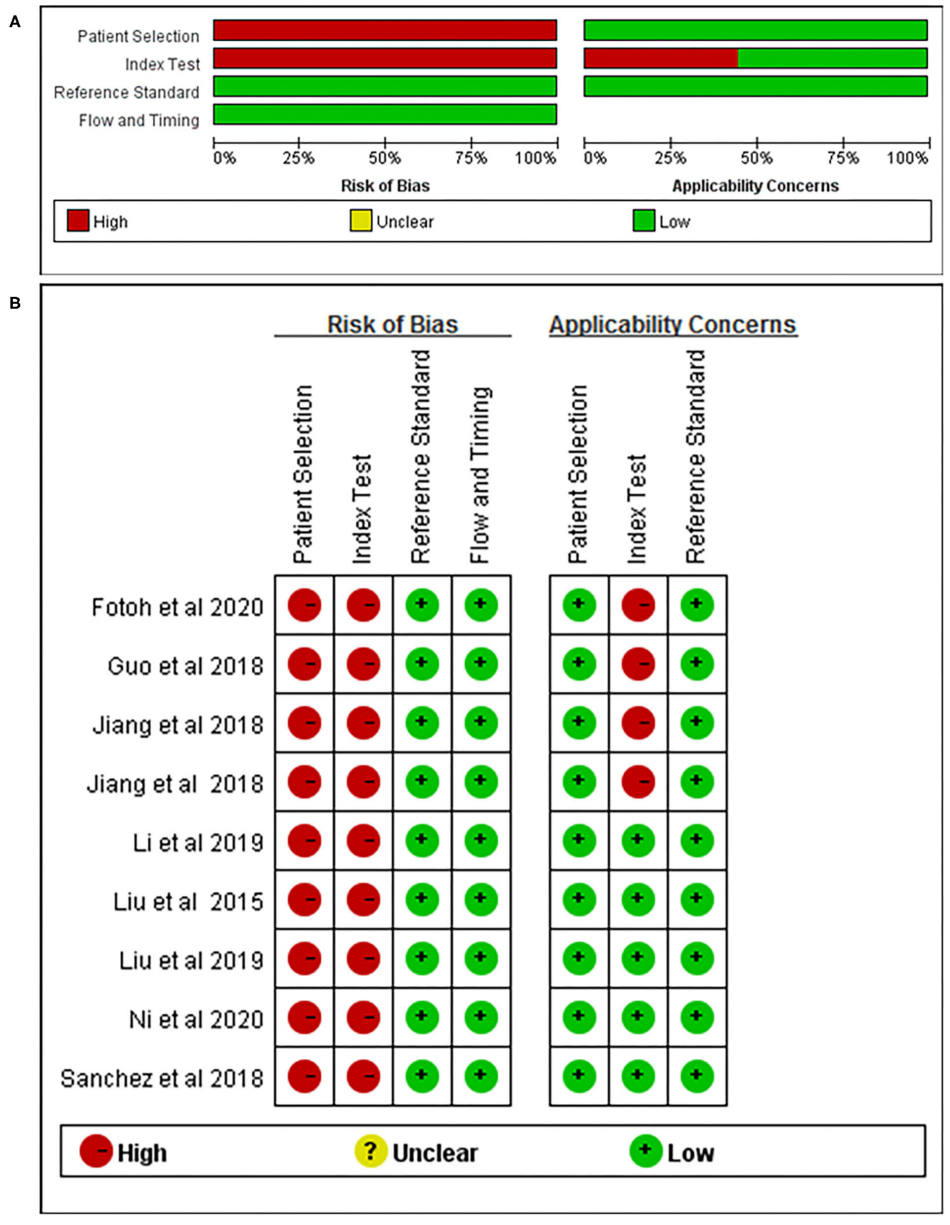
QUADAS-2 assessment. Risk of bias and applicability concerns' **(A)** graph and **(B)** summary.

Nine articles that reported on the diagnostic accuracy of AS were included in the systematic review (Table 2). In total, the nine articles (time period ranging from years of 2015 to 2020) reported 29 studies, including 496 AS patients and 383 controls. Among the 29 included studies, one study detected miRNA in whole blood, two studies detected miRNA in blood serum, 13 studies researched on blood plasma, and 13 studies researched peripheral blood mononuclear cells (PBMCs). Among the 29 studies, 21 studies were conducted in Chinese populations, six studies were conducted in Spanish populations, and the remaining two studies focused on Egyptian populations. The quality of the included articles was assessed using the modified version of the QUADAS-2 tool. The risk of bias and applicability concerns graph for the included articles is presented in Figure 2.

**Table 2:** Characteristics of the nine reports in our meta-analysis.

**Study**	**Year**	**Country**	**Patients/controls**	**miRNAs profiled**	**Sensitivity (%)**	**Specificity (%)**	**AUC**	**Specimen**
Liu et al. (17)	2020	China	32/24	miR-214	78.13	96	0.8625	Blood (serum)
Ni and Leng (18)	2020	China	150/150	miR-495	85	49	0.6052	Blood (serum)
				miR-495	98	53	0.6576	Whole Blood
				miR-495	63	61	0.7849	PBMCs
Perez-Sanchez et al. (19)	2018	Spain	53/57	miR-22-3p	55.3	74.5	0.732	Blood (plasma)
				miR-146a-5p	60.4	77.2	0.687	
				miR-125a-5p	51	78.9	0.751	
				miR-151a-3p	61.2	79.6	0.781	
				miR-451a	51	61.4	0.622	
				miR-150-5p	50.9	64.7	0.614	
Li et al. (20)	2019	China	43/39	miR-17-5p	56.5	89.7	0.78	PBMCs
				miR-27a	56.5	97.4	0.758	
				miR-29a	91.3	92.3	0.952	
				miR-126-3p	75.4	82.1	0.852	
Guo et al. (21)	2018	China	218/113	miR-132	91.7	97.3	0.965	PBMCs
Jiang et al. (22)	2018	China	60/60	miR-17	83.33	86.67	0.982	PBMCs
				miR-17	83.33	86.67	0.964	Blood (plasma)
Jiang et al. (23)	2018	China	60/30	miR-146a	85.00	95.33	0.916	Blood (plasma)
				miR-155	80.00	83.33	0.901	
				miR-181a	85.00	86.67	0.939	
				miR-223	61.67	56.67	0.629	
				miR-146a	77.33	76.67	0.795	PBMCs
				miR-155	75.00	80.00	0.837	
				miR-181a	83.33	80.00	0.870	
				miR-223	71.67	76.67	0.821	
Liu (24)	2015	China	61/31	miR-49	92.23	58.06	0.729	PBMCs
				miR-181	76.67	54.84	0.697	
Fotoh et al. (25)	2020	Egypt	55/55	miR-125a	70.91	83.64	0.788	Blood (plasma)
				miR-451a	64.45	78.18	0.802	

*PBMCs, peripheral blood mononuclear cells; SEN, sensitivity; SPE, specificity; AUC, area under the curve*.

### Diagnostic Accuracy

**Figure 3:**
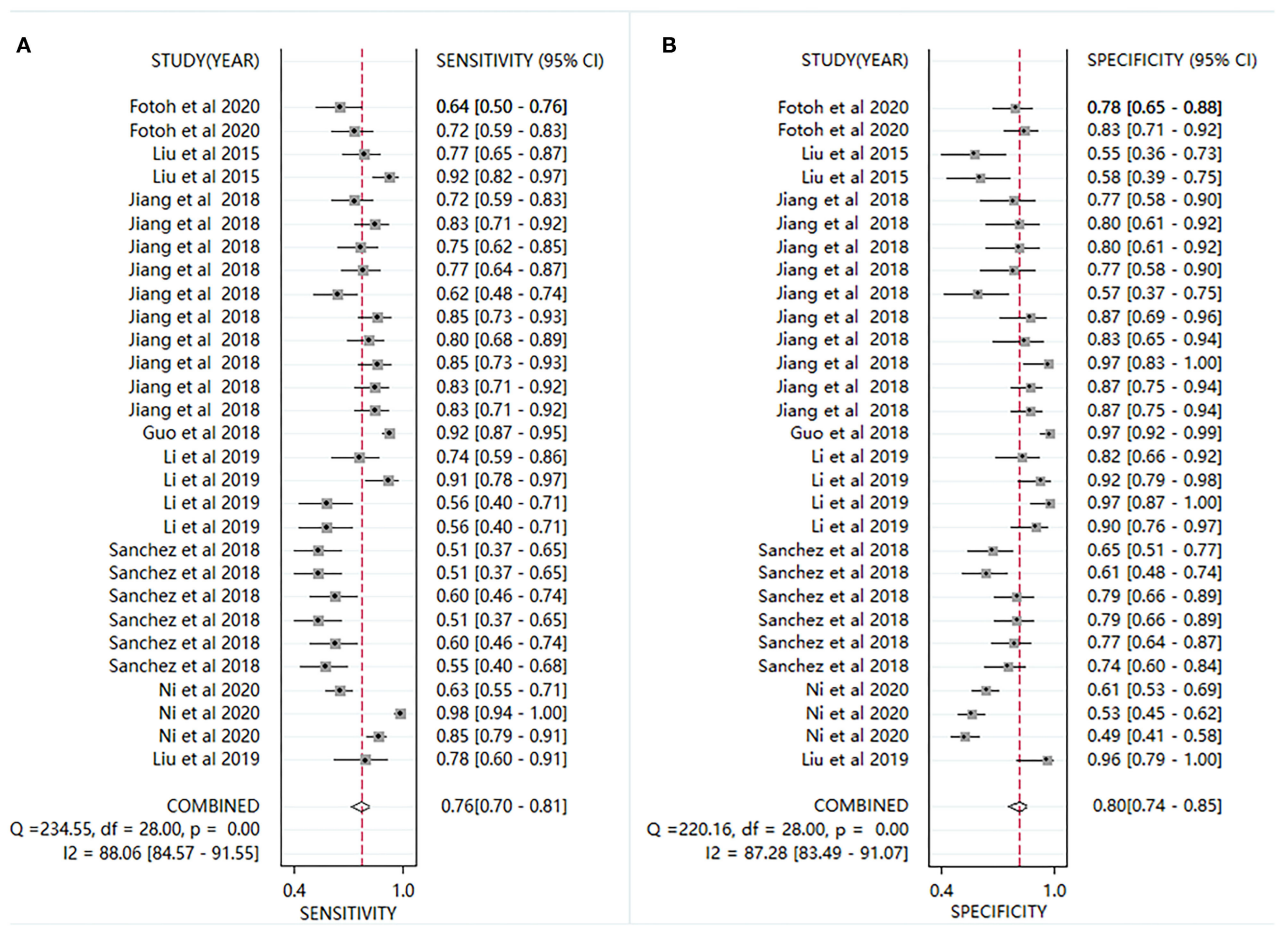
Forest plots for studies on individual miRNA used in the diagnosis of AS among the 29 studies included in the meta-analysis. **(A)** sensitivity; **(B)** specificity.

**Figure 4:**
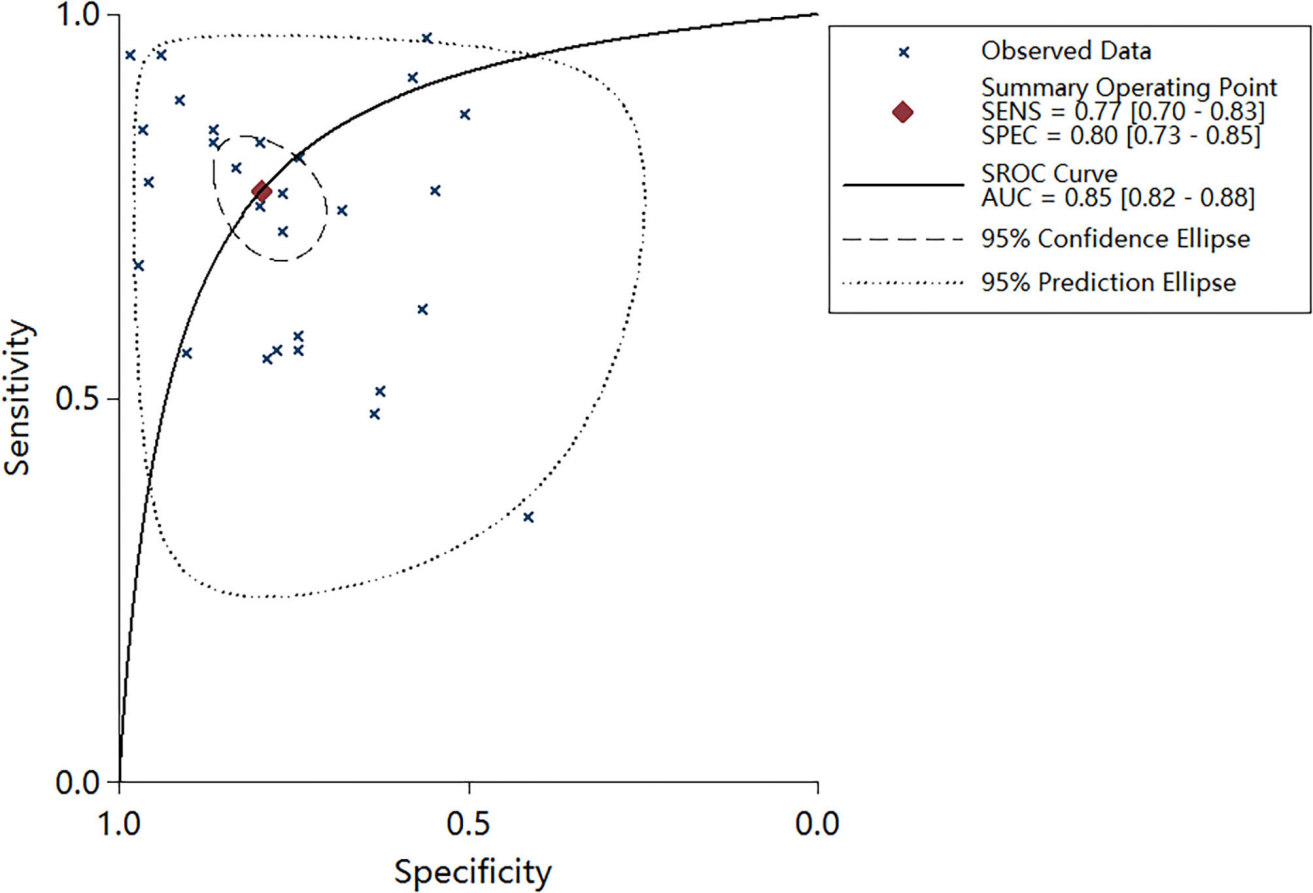
Summary receiver operator characteristic (SROC) curve with area under curve (AUC) based on miRNAs.

Plots of sensitivities and specificities are shown in Figure 3. The results of the *I*^2^ test were 88.06% for sensitivity and 87.28% for specificity, which suggested that there was significant heterogeneity between the results of the included studies, and thus the random-effects model was selected. The sensitivity [±95% confidence intervals (CIs)] and specificity (±95% CIs) for each miRNA are shown in the corresponding forest plots (Figures 3A,B). The overall sensitivity and specificity of the 29 individual miRNAs in the diagnosis of AS were 0.76 (95% CI, 0.70–0.81), and 0.80 (95% CI, 0.74–0.85), respectively. The SROC curve is shown in Figure 4, with AUC of 0.85 (95% CI, 0.81–0.88). The overall PLR and NLR are shown in Table 3, the pooled PLR and NLR were 3.75 (95% CI, 2.82–5.01), 0.30 (95% CI, 0.24–0.39), These results indicate good discriminative ability of using miRNAs as biomarkers for the detection of AS.

**Table 3:** Diagnostic performance of miRNAs in AS patients.

**Analysis**	**Overall**	**Chinese**	**Spanish**	**PBMCs**	**Blood (plasma)**
No. of studies	29	21	6	13	12
SEN (95% CI)	0.76 (0.70–0.81)	0.81 (0.75–0.86)	0.55 (0.49–0.60)	0.78 (0.71–0.84)	0.67 (0.59–0.75)
*I*^2^, % (95% CI)	88.06 (84.57–91.55)	86.97 (82.36–91.59)	0.00 (0.00–100.00)	85.69 (79.00–92.38)	78.47 (67.23–89.72)
SPE (95% CI)	0.80 (0.74–0.85)	0.82 (0.74–0.88)	0.73 (0.67–0.78)	0.83 (0.74–0.90)	0.79 (0.73–0.84)
*I*^2^, % (95% CI)	87.28 (83.49–91.07)	90.28 (87.12–93.44)	39.22 (0.00–95.38)	87.70 (82.18–93.21)	68.51 (50.39–86.64)
PLR (95% CI)	3.75 (2.82–5.01)	4.52 (3.06–6.70)	2.01 (1.56–2.57)	4.65 (2.87–7.52)	3.22 (2.23–4.64)
*I*^2^, % (95% CI)	82.73 (82.73–90.73)	86.77 (86.77–93.30)	0.00 (0.00–95.94)	84.68 (84.68–93.90)	69.73 (69.73–90.24)
NLR (95% CI)	0.30 (0.24–0.39)	0.23 (0.18–0.31)	0.62 (0.53–0.73)	0.26 (0.19–0.36)	0.41 (0.31,0.55)
*I*^2^, % (95% CI)	88.34 (84.95–91.72)	87.49 (83.11–91.87)	13.92 (0.00–100.00)	88.33 (83.18–93.48)	84.26 (76.70–91.81)
DOR (95% CI)	12.32 (7.65–19.83)	19.50 (11.08–34.31)	3.22 (2.17–4.78)	17.80 (8.70–36.40)	7.81 (4.10–14.89)
AUC	0.85 (0.81–0.88)	0.88 (0.85–0.91)	0.62 (0.58–0.66)	0.87 (0.84–0.90)	0.80 (0.77–0.84)

*PBMCs, peripheral blood mononuclear cells; CI, confidence interval; SEN, sensitivity; SPE, specificity; PLR, positive likelihood ratio; NLR, negative likelihood ratio; DOR, diagnostic odds ratio; AUC, area under the curve*.

### Subgroup Analysis and Meta-Regression Analysis

To explore the between-study heterogeneity in this meta-analysis, we carried out subgroup analyses and meta-regression analysis based on ethnicity and detected sample. As shown in Table 3, *I*^2^ > 50% of each parameter indicated significant heterogeneity and thus the random-effects model was selected. However, ethnicity and sample types aren't the sources of heterogeneity in sensitivity and specificity. The pooled sensitivity, specificity, DOR, and AUC were 0.76 (95% CI, 0.70–0.81), 0.80 (95% CI, 0.74–0.85), 12.316 (7.651–19.826), 0.85 (0.81–0.88), respectively. In detected samples of peripheral blood mononuclear cells (PBMCs), the results were 0.78 (0.71–0.84) for sensitivity, 0.83 (0.74–0.90) for specificity, 4.65 (2.87–7.52) for PLR, 0.26 (0.19–0.36) for PLR, 17.80 (8.70–36.40) for DOR, and 0.87 (0.84–0.90) for AUC. In the blood plasma samples, the sensitivity, specificity, PLR, NLR, DOR, and AUC was 0.67 (0.59–0.75), 0.79 (0.73–0.84), 3.22 (2.23–4.64), 0.41 (0.31–0.55), 7.81 (4.10–14.89), and 0.80 (0.77–0.84), suggesting that miRNAs in PBMCs, rather than in blood plasma, has a higher diagnostic accuracy. Furthermore, the sensitivity, specificity, PLR, and NLR for Chinese were 0.81 (95% CI, 0.75–0.86), 0.82 (95% CI, 0.74–0.88), 4.52 (95% CI, 3.06–6.70), and 0.23 (95% CI, 0.18–0.31), respectively, with a DOR of 19.50 (95% CI, 11.08–34.31) and AUC value of 0.88 (95% CI, 0.85–0.91). Compared with the Spanish, miRNAs have a higher overall diagnostic accuracy in the Chinese, and they have important diagnostic value for AS disease in the Chinese. Meta-regression was further conducted to explore the potential sources of the interstudy heterogeneity in sensitivity and specificity. Ethnicity were not the source of heterogeneity in sensitivity (*P* = 0.07) and specificity (*P* = 0.21). Besides, the specimen types were also not the source of heterogeneity in sensitivity (*P* = 0.57) and specificity (*P* = 0.71).

### Publication Bias

**Figure 5:**
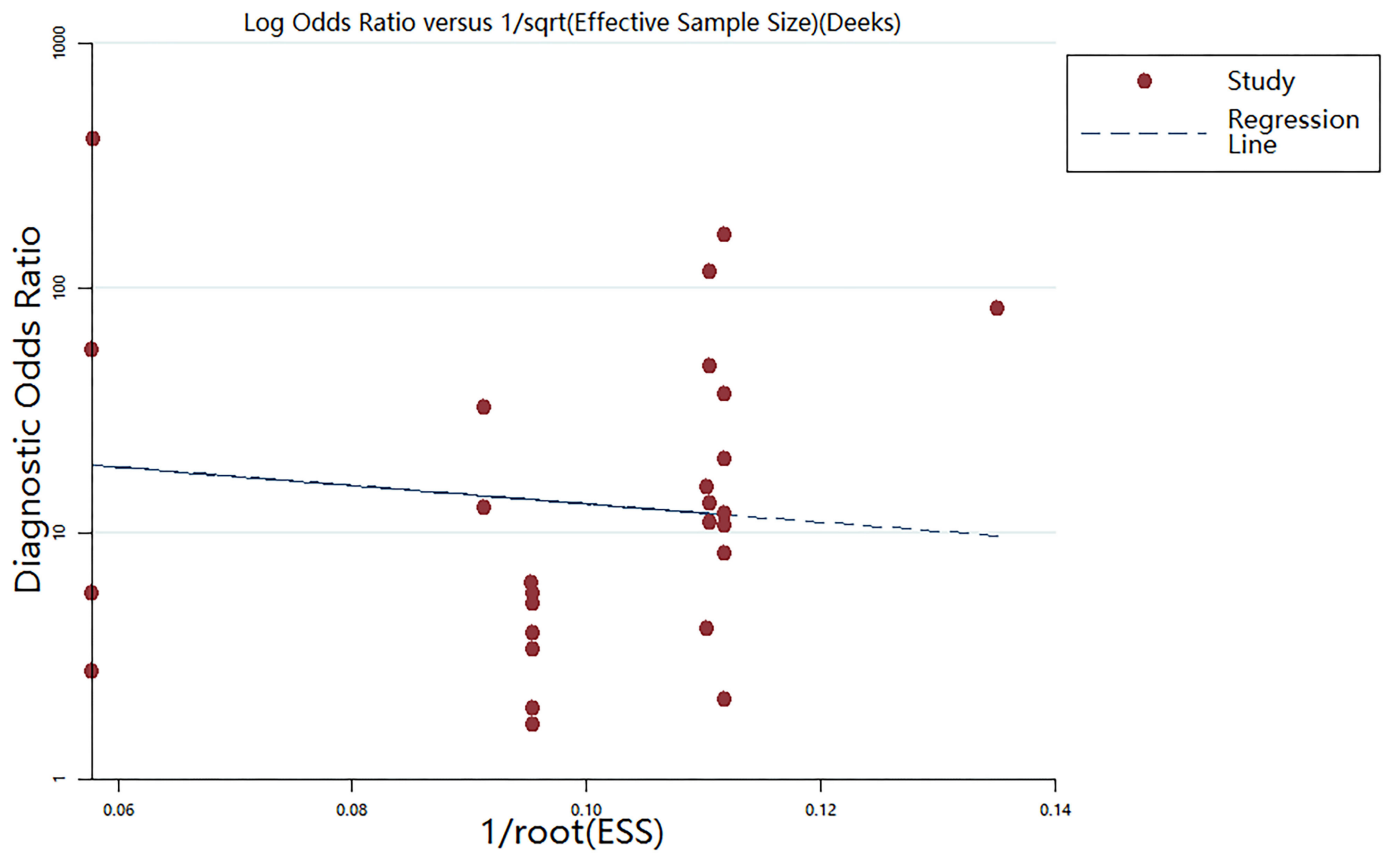
The Deeks' funnel plot asymmetry test (*P* = 0.144).

The Deeks' funnel plot asymmetry test was performed (Figure 5). The *P*-value of 0.144 suggested that no bias existed in the included publications.

## Discussion

To date, the early diagnosis of AS is still challenging, because the pathogenesis of AS remains unclear. Besides, X-ray fails to detect early radiological changes of AS. As a result, the diagnosis of AS is often delayed, even up to 7–11 years after the onset of the symptoms (45, 46). Currently the biomarkers such as HLA-B27, erythrocyte sedimentation rate, and C-reactive protein only have moderate diagnostic value, and their sensitivity and specificity are suboptimal (47). On the contrary, substantial progress has been made in improving the early diagnosis for other rheumatic diseases, such as rheumatoid arthritis (RA) (48). Novel biomarkers for the early diagnosis of AS are urgently needed. Over the past years, miRNAs have been observed an important role in the pathologies of systemic rheumatic diseases (49). miRNAs are actively involved in the deregulation of immune cell functions, regulation of T-cell survival signaling, and proinflammatory pathway. In addition, bone remodeling is controlled through miRNAs, such as miR-29a, miR-21, miR-34a, miR-17-5p, and miR-27b-3p. miRNAs, such as miR-214, also control T-cell autophagy, which might be involved in the pathological process of AS (50). Circulating miRNAs are promising diagnostic biomarkers for AS because of their high sensitivity and specificity for AS. Besides, they are relatively stable and can be easily detected in blood serum or plasma, which allows repeated sampling to monitor the development of the disease (51). Since the first AS-associated miRNAs was discovered in blood (37), there are increasing number of articles reporting on their expression in recent years. Thus, we conduct this research to evaluate the overall diagnostic accuracy of miRNAs in AS patients.

This systematic review has identified 87 miRNAs which were differentially expressed between AS and healthy controls in these screened articles, and of which, 42 miRNAs were observed to be up-regulated and 45 miRNAs were down-regulated in AS cases compared with the controls. Twelve up-regulated miRNAs (miR-29, miR-146a, miR-126-3p, miR-155, miR-21, miR-214, miR-16, miR-181a, miR-223, miR-146a-5p, miR-151a-3p, and let 7i) and two down-regulated miRNAs (miR-17, miR-495) were identified by more than one studies, and miR-29 was the most frequently identified dysregulated miRNA. Besides, miR-29 was found to be either up-regulated or down-regulated in AS cases compared with controls. Disease activity and therapy may be the sources of heterogeneity about the expression of miR-29. The expression of miR-29a was significantly lower in active AS patients than in controls. However, miR-29a was significantly upregulated in AS patients after 12-week of etanercept therapy than that in controls. When there was a good therapeutic effect, the expression of the miRNAs would get up-regulated and tend to be normal (38). On the contrary, plasma miR-22–3p, miR-146a-5p, and miR-125a-5p expression levels were significantly up-regulated in active AS patients when compared with non-active AS patients (19). miR-29a, miR-22–3p, miR-146a-5p, and miR-125a-5p will probably become the potential biomarkers, activity evaluation, and the curative effect monitoring of AS. miR-29a is part of the miR-29 family (miR-29a, miR-29b, and miR-29c), which are expressed in both T- and B-cells, as well as dendritic cells. It has been identified that miR-29a plays an important role in the regulator of immunity (52) and the bone metabolism, and miR-29a can modulate Wnt signaling to accelerate bone formation (53). Interestingly, miR-29c, another member of the miR-29 family, was observed to be down-regulated in AS patients compared with controls in two studies (26). Additionally, six miRNAs (miR-214, miR-223, miR-151a-3p, miR-146a-5p, miR-146a, and miR-16) were observed to be either up-regulated or down-regulated in the AS cases as compared with the controls. Inconsistent results about dysregulated miRNAs may be caused by a variety of factors, including ethnic differences, the severity of the disease, as well as the source of samples, and sample sizes. In the present meta-analysis, a pooled sensitivity of 0.76 (95% CI, 0.70–0.81), specificity of 0.80 (95% CI, 0.74–0.85), PLR of 3.75 (95% CI, 2.82–5.01), NLR of 0.30 (95% CI, 0.24–0.39), DOR of 12.32 (7.65–19.83) and AUC of 0.85 (95% CI, 0.81–0.88) demonstrate that as a diagnostic biomarker, circulating miRNAs achieved a relatively high overall accuracy in diagnosing AS. However, also as a diagnostic biomarker for AS, its serum CRP has only 50% sensitivity and 80% specificity (54). In addition, we carried out subgroup analyses to explore the sources of heterogeneity. Compared with the Spanish, miRNAs have a higher overall diagnostic accuracy in Chinese. This result may be related to the sample size of the reported articles, because we eventually only included 53 patients from one article about the miRNAs expression profile of Spanish. However, 624 patients from seven articles about the miRNAs expression profile of Chinese were included. Besides, miRNAs in PBMCs showed higher diagnostic value than in blood plasm. The expression of miRNAs in PBMCs is likely to be more stable than that in blood plasm. Thus, for a better blood-based biomarker for AS, miRNAs in PBMCs is preferred.

The current meta-analysis is the first ever to evaluate the overall diagnostic accuracy of miRNAs for AS as to our knowledge. We combined the results of 29 studies with 732 patients and 559 controls, which may enhance the statistical power and the reliability of the findings. Because the study's heterogeneity (*I*^2^ 88.06% for sensitivity, and 87.28% for specificity) was significant, we adopted the random effects model to reduce the heterogeneity related bias. We found that miRNAs in PBMCs, rather than in blood plasma, had a higher diagnostic accuracy for AS. However, meta-regression and subgroup analysis didn't find the sources of heterogeneity, suggesting that complex factors influence the final results. There is almost no publication bias by the Deeks' funnel plot asymmetry test, strengthening the reliability of the results. We not only reviewed the pooled diagnostic accuracy of circulating miRNAs, but also systematically summarized the diagnostic effectiveness of every individual miRNA, which provide valuable reference data for further research. However, the present paper has some limitations. First, due to the different kinds of miRNAs, small sample size, and different cut-off values in the selected articles, significant heterogeneity was existed in the current meta-analysis, which is a common situation in other published meta-analyses of diagnostic accuracy. Moreover, the overall diagnostic accuracy may be amplified, because those articles with positive results are more likely to be published. In addition, the biological characteristics and mechanisms of the different miRNAs in AS may differ, which may limit the applicability of the pooled analysis. It is expected that in the future more studies from different genetics, geography, and ethnicity may be carried out to increase the validity of our conclusions and the reliability of miRNA analysis for AS.

## Conclusion

In summary, we identified numerous circulating miRNAs associated with AS in this systematic review. The circulating miRNAs may be used as biomarkers for the detection of AS. It was also found that miRNA in PBMCs, rather than in blood plasma, has a higher diagnostic accuracy. The diagnosis of miRNAs for AS seems to be more sensitive in Chinese than that in Spanish. Further studies based on larger sample of patients and controls are still required.

## Data Availability Statement

The original contributions presented in the study are included in the article/Supplementary Material, further inquiries can be directed to the corresponding author.

## Author Contributions

JL and XX designed the study and collected data. JL drafted the manuscript. WL, FG, KZ, ZSu, QW, ZSui, and PZ contributed to the writing. TY provided critical feedback and contributed to the review of the manuscript. All authors contributed to the article and approved the submitted version.

## Conflict of Interest

The authors declare that the research was conducted in the absence of any commercial or financial relationships that could be construed as a potential conflict of interest.

## Publisher's Note

All claims expressed in this article are solely those of the authors and do not necessarily represent those of their affiliated organizations, or those of the publisher, the editors and the reviewers. Any product that may be evaluated in this article, or claim that may be made by its manufacturer, is not guaranteed or endorsed by the publisher.
