# Supplementary material for: MicroRNAs as Biomarkers for the Diagnosis of Ankylosing Spondylitis: A Systematic Review and Meta-Analysis
Source: Front Med (Lausanne). 2021 Aug 10;8:701789. doi: 10.3389/fmed.2021.701789 (PMC8383110; doi:10.3389/fmed.2021.701789)
Supplement: Supplementary file 1 [file Table_1.DOCX]

Supplementary Table 1. Summary of search strategies.

| **Pubmed** | | |
| --- | --- | --- |
| Search | Query | Results |
| #1 | (((((MicroRNAs[MeSH Terms]) OR (MicroRNA[Title/Abstract])) OR (miRNA[Title/Abstract])) OR (RNA, Micro[Title/Abstract])) OR (Micro RNA[Title/Abstract])) OR (Primary MicroRNA[Title/Abstract]) | 120134 |
| #2 | (((((Spondylitis, Ankylosing[MeSH Terms]) OR (Spondyloarthritis Ankylopoietica[Title/Abstract])) OR (Ankylosing Spondylarthritis[Title/Abstract])) OR (Ankylosing Spondylarthritides[Title/Abstract])) OR (Spondylarthritides, Ankylosing[Title/Abstract])) OR (Ankylosing Spondylitis[Title/Abstract]) | 19897 |
| #3 | ((blood[MeSH Terms]) OR (serum[Title/Abstract])) OR (plasma[Title/Abstract]) | 2812130 |
| #4 | #1 AND #2 AND #3 | 33 |
| **Embase** | | |
| #1 | (MicroRNAs[Title/Abstract]) OR (MicroRNA[Title/Abstract]) OR (miRNA[Title/Abstract]) OR (RNA, Micro[Title/Abstract]) OR (Micro RNA[Title/Abstract]) OR (Primary MicroRNA[Title/Abstract]) | 140170 |
| #2 | (Spondylitis, Ankylosing[Title/Abstract]) OR (Spondyloarthritis Ankylopoietica[Title/Abstract]) OR (Ankylosing Spondylarthritis[Title/Abstract]) OR (Ankylosing Spondylarthritides[Title/Abstract]) OR (Spondylarthritides, Ankylosing[Title/Abstract]) OR (Ankylosing Spondylitis[Title/Abstract]) | 24695 |
| #3 | (blood[Title/Abstract]) OR (serum[Title/Abstract]) OR (plasma[Title/Abstract]) | 4550611 |
| #4 | #1 AND #2 AND #3 | 54 |
| **Cochrane Library** | | |
| #1 | (MicroRNAs[Title/Abstract/Keyword]) OR (MicroRNA[Title/Abstract/Keyword]) OR (miRNA[Title/Abstract/Keyword]) OR (RNA, Micro[Title/Abstract/Keyword]) OR (Micro RNA[Title/Abstract/Keyword]) | 1292 |
| #2 | (Spondylitis, Ankylosing[Title/Abstract/Keyword]) OR (Spondyloarthritis Ankylopoietica[Title/Abstract/Keyword]) OR (Ankylosing Spondylarthritis[Title/Abstract/Keyword]) OR (Ankylosing Spondylarthritides[Title/Abstract/Keyword]) OR (Ankylosing Spondylitis[Title/Abstract/Keyword]) | 431637 |
| #3 | (blood[Title/Abstract/Keyword]) OR (serum[Title/Abstract/Keyword]) OR (plasma[Title/Abstract/Keyword]) | 2181 |
| #4 | #1 AND #2 AND #3 | 3 |
| **Wan Fang databases** | | |
| #1 | (MicroRNAs[Title/Abstract/Keyword]) OR (MicroRNA[Title/Abstract/Keyword]) OR (miRNA[Title/Abstract/Keyword]) OR (RNA, Micro[Title/Abstract/Keyword]) OR (Micro RNA[Title/Abstract/Keyword]) | 57772 |
| #2 | (Spondylitis, Ankylosing[Title/Abstract]) OR (Ankylosing Spondylitis[Title/Abstract/Keyword]) | 16885 |
| #3 | (blood[Title/Abstract/Keyword]) OR (serum[Title/Abstract/Keyword]) OR (plasma[Title/Abstract/Keyword]) | 554000 |
| #4 | #1 AND #2 AND #3 | 27 |
